# Cryo-Imaging of Hydrogels Supermolecular Structure

**DOI:** 10.1038/srep25495

**Published:** 2016-05-05

**Authors:** Clement Marmorat, Arkadii Arinstein, Naama Koifman, Yeshayahu Talmon, Eyal Zussman, Miriam Rafailovich

**Affiliations:** 1 Department of Materials Science and Engineering, Stony Brook University, New York, USA; 2 Department of Mechanical Engineering, Technion, Haifa, Israel; 3 The Wolfson Department of Chemical Engineering, Technion, Haifa, Israel

## Abstract

Gelatin, derived from collagen, has both the mechanical properties required for tissue growth, as well the functional domains required for cell binding. In its natural state, gelatin derives its properties from a network of structured, intertwined, triple helical chains, which is stabilized by hydrogen bonds at temperatures below 37 °C. The mechanical properties of such a structure can be further controlled by additional enzymatic cross-linking. But, in contrast to simple polymer systems, the response to an imposed deformation is here determined by two competing factors: the establishment of the cross-linked mesh vs. the self-assembly of the fibrils into larger and stronger hierarchical structures. Therefore, properties deduced from the response to measurements such as rheology or swelling, are a combination of these two very different factors, hence a modeling is impossible unless more precise knowledge regarding the internal structure is available. The cryogenic-temperature scanning electron microscopy (cryo-SEM) was adopted to image the fully hydrated gelatin network in which distinct chain folding was observed at low densities, while cross-linked networks were observed at higher densities. Based on these images, a theoretical model which results in good agreement between the mesh sizes of both networks and their mechanical properties was developed.

Gelatin is a naturally derived, easily accessible, and cheap material which was studied extensively in the last decade as it was used in numerous applications, ranging from food to tissue engineering^1^. Gelatin consists of cleaved collagen proteins which present a partially coiled, fibril like network at temperatures below 37 °C. The unique structure of gelatin arises due to a combination of Van der Waals interactions and hydrogen bonds which become unstable above 37 °C, when thermal fluctuations cause the chains to uncoil and form a homogeneous solution^2^,^3^. Therefore, in order to use gelatin as a structural component in a biocompatible scaffold, the network must be stabilized against dissolution at physiological temperatures^4^. This can be accomplished via enzymatic cross-linking, using Microbial Transglutaminase (MTG) which forms an iso-peptide bond between the amine groups of a lysine amino acid with the acyl group of a glutamine amino acid as seen on Fig. 1 Upon reaction with MTG in solution at temperatures higher than 37 °C, neighbor gelatin chains will be covalently bound to one another and will create a permanent network of gelatin strands^5^,^6^.

However, in such systems the duality in bonding nature creates a complicated system at room temperature, where the hydrogel strength derives from two different factors: the presence of stable covalently bonded gelatin strands competing with their natural tendency of re-coiling into fibrils^7^. In contrast to a simple cross-linked network, the interplay between these two structures determines the rheological response of complex structures such as gelatin or collagen. Unfortunately, these structures are hard to observe once the gel is dehydrated and often coated, and standard SEM microscopy cannot be applied to the swollen gel. Therefore, in order to directly observe these features we developed an imaging method of frozen fractured hydrogel samples by cryo-SEM microscopy^7^,^8^ where the obtained images were sufficiently detailed for direct comparison with theory.

Gelatin solution was synthetized by dissolving type A gelatin at a concentration of 10% (w/v) into deionized water, and heating the solution at 65 °C for 15 minutes until complete dissolution was obtained. We used two different concentrations of MTG enzyme to cross-link the hydrogels. High and low cross linking density formulations were prepared by adding 3.8 mg.ml^−1^ and 0.5 mg.ml^−1^ of MTG at 42 °C to the gelatin solution, and incubated for 24 hours at 37 °C to allow cross-linking to occur. The samples were kept tightly capped in order to avoid water evaporation and volume changes during the reaction. After 24 hours the gels were swollen at room temperature with excess water to reach equilibration of the network water-full porosities.

Rheology was first performed to assess the differences between the two gels in terms of mechanical properties^7^ (see Fig. 2a). The gels were then swollen at room temperature in water for 24 hours after cross linking in order to reach their equilibrated state. One can see that the moduli differ by approximately one order of magnitude at the cross-linking temperature and below (see Fig. 2a), which is not surprising, since it scales with the difference in MTG concentration responsible for the cross-linking reaction. The combination of hydrogen bonding and cross-linking density in determining the internal structure of the gels is illustrated above (see Fig. 2b,c). In these Figures we show that the structure of the gel is a compromise between a network formed by chemical cross-links, and the physical association between protein strands due to hydrogen bonding at temperatures below 37 °C. Thus, a highly ordered self-assembled lattice competes with the fluctuating random network produced by the cross-linking enzymes. At temperatures above 37 °C, the least cross linked gels exhibits a severe decrease of its elasticity parameter which is related to the instability of the hydrogen-bonded coiled natural structure of gelatin at these temperatures. This hypothesis is illustrated in Fig. 2 where one can see that for the softly cross-linked gel, the distance between cross-links is sufficiently large to allow, upon cooling, the self-assembly of the chain segments into the natural helical configuration which then co-exists at room temperature with the cross-linked network. Hence, the gel modulus below the self-assembly transition may be harder than expected, if only the degree of cross-linking was involved. In the case of the highly cross-linked gel, the chains are confined by the cross linking regions which prevents any other reorganization upon cooling. Therefore, this configuration would not show a significant change in modulus with temperature. Note that the rheology measurement alone is insufficient to determine the structure, and by this reason we performed cryo-SEM in the hope that we could actually image the lattice^9^.

Both soft and hard hydrogels were synthetized following the same procedure as mentioned above. The samples were rapidly cooled and fractured from room temperature using the method developed by Talmon’s group^8^ and adapted to our system, as described in the supplementary material. Fast cooling minimized freezing expansion and preserved fine nanoscale details of the nanostructure by vitrifying the water swollen in the network, which can then be directly imaged by SEM at cryogenic temperatures, typically below −145 °C. The samples showed, as expected, unnoticeable geometrical expansion during the procedure. The images thus obtained are shown in Fig. 3, where we can see that both low and high MTG concentrations produce cross-linked structures which are swollen by the water phase. Scanning of the beam resulted in partial etching of the sample and lead to artificial reorganization of the hydrogels surfaces (see supplementary material Figures S4 and S5).

Comparison of the images in Fig. 3 clearly shows that the network mesh size is smaller when the cross-linking density is higher, as expected. The structure of the networks, though, is fundamentally different. The highly cross-linked network is composed of interconnected single strands of gelatin creating a mesh of an average size of *ξ*_*obs.hard*_ ≈ 342 ± 72 nm (see supplementary material Table S2). The network with the low cross linking density forms a much more complex structure composed of very large highly ordered fibers which branch at angles of approximately 120 ± 30 degrees (see supplementary material Table S3). Closer examination of the fibers (see insert in Fig. 3b) shows that each is composed of a secondary structure, with a well-defined banded pattern with a periodicity of 64 nm. This pattern is well known in collagen, where the molecules self-assemble in a staggered formation with period of 64 nm^10^. These large fibers are consistent with the model proposed above (Fig. 2b,c). The larger cross-linking period, of a mesh size *ξ*_*obs.soft*_ ≈ 873 ± 284 nm (see supplementary Table S2) is consistent with the lower cross-linking density, as shown. Upon cooling, the large spacing between cross-linking points allows the fiber sufficient mobility to rearrange into the characteristic fibril-like structure of collagen at *T* < 37 °C. The resulting fiber bundles are much stronger, and hence provide stiffness to the network resulting in the angular pattern shown.

Cross-linked polymer or protein gels are currently modeled using standard rubber elasticity theory (RET)^11^, which relates the mesh size, *ξ*, to the modulus, *G*′, with the well-known equation:


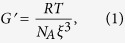


here *N*_*A*_ is the Avogadro number, *R* is the perfect gas constant and *T* is the temperature.

Substituting the elastic modulus values obtained from the rheology measurements of the highly cross-linked gel at room temperature (Fig. 2a), into equation (1), we obtain that *ξ* ≈ 6.6 nm which is similar to the values previously quoted by numerous authors using RET analysis.

Swelling experiments can also be performed to evaluate the mesh size of semi-flexible polymeric networks^12^,^13^,^14^,^15^,^16^,^17^. In this case, the equilibrium stress in the network arises due to a solvent and the corresponding strain is derived by using the Flory-Rhener model^18^ which, in turn, is a modified form of RET which includes the polymer-solvent interaction. In order to compare with the values obtained from the equation (1), once cross-linked, the gels were swollen in deionized water. After recording the gels mass, gelatin volume fraction, 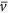, in the network was derived as follows:


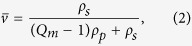


here *ρ*_*p*_ and *ρ*_*s*_ are the density of the polymer and of the solvent, respectively; *Q*_*m*_ = *M*_*sw*_/*M*_*p*_ is the swelling ratio; and *M*_*sw*_ and *M*_*p*_ are the masses of the swelled and dry samples, respectively.

The polymer volume fraction, 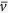, can then be used to determine the molecular weight between crosslinks in a non-ionized gel with a Gaussian distribution of the polymer chains:


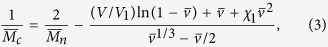


here 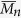 is the average molecular weight of the polymer, *V*_1_ is the molar volume of the solvent, *χ*_1_ is the Flory Huggins interaction parameter, and *V* is the specific volume of the polymer.

Finally, we have to consider the gelatin strands persistence length as well as its contour length to calculate the mesh size of the fully cross-linked network:


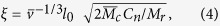


here 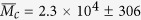 is the calculated average molecular weight between two neighbor crosslinks; *M*_*r*_ is the molecular weight of the repeating unit; *l*_0_ is the length of the bond along the polymer backbone which can be derived from the arithmetic mean of one C-C bond and two C-N bonds to be about 1.4 Å^19^, *C*_*n*_ is the characteristic ratio of the polymer which can be calculated as:


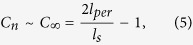


where *l*_*per*_ is the persistence length of the gelatin, and *l*_*s*_ is the linear segment length, here *l*_*s*_ = *l*_*0*_.

From this equation (5) we obtain that *ξ* ≈ 21.3 ± 0.22 nm, (See supplementary material Table S1).

In our case, we can directly compare this value with the microscopic image, and we find that, even though it is three times larger than the RET value, it still underestimates the actual value by one order of magnitude. Indeed, RET assumes that the mesh is composed of fully flexible polymer chains interconnected by tetragonal junction types. On the other hand, the Flory-Rhener model is only accurate in the case of perfectly distributed polymer chains composing the cross-linked network with swelling behavior depending on the equilibrium between thermodynamic compatibility of the polymers and the solvent, and retractile force due to extensive stretching of the network cross-links^20^. Swelling experiments provide an increased value for the mesh size estimation, but it remains still under-estimated^18^,^21^ (Fig. 3a). In a bio polymeric system that presents bundles or coiled secondary structures the higher rigidity of the segments between the cross-linked junctions needs to be considered^22^.

In general, the elastic modulus, *G*′, of a network consisting of effective springs with effective elastic constant, *k*_*ef*_, can be estimated as:


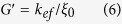


here *ξ*_0_ is the network mesh size.

The partially cross-linked network can be considered as an arrangement of large gelatin bundles interconnected by individual gelatin strands (see Fig. 4a).

Assuming that the bundles are hard elements (no stretching and no bending of the fibrils are possible), and the tie molecules are semi-flexible and non-stretchable, we have to conclude that the system stretching can only occur due to a straightening of α angle between the connecting tie molecules (see Fig. 4b).

The straightening of the tie molecules under external action (stretched force, *F*_*ext*_) can be considered as an elongation of a spring with effective elastic constant, *k*_*ef*_, in accordance with the Hook’s law: *F*_*ext*_ = *k*_*ef*_*δL*. The effective elastic constant of the spring presenting a tie molecule, is a function of the bending rigidity, *a*, which determines the persistence length, *l*_*per*_ ∝ *a*/*k*_*B*_*T*. In the case of semi-flexible gelatin sub-chains, the effective elastic constant, *k*_*ef*_, can be calculated similarly to calculations of Treolard *et al.*, derived for PS polymer chains^23^:


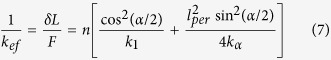


*k*_1_ being the elastic constant of the chain along the polymer backbone direction and *k*_*a*_ the one across that direction. Here we can assume that *k*_1_ is very large, and <sin^2^(*α/*2)> is not too small, we can omit the first term in equation (7), so the effective elastic constant, *k*_*ef*_, can be estimated as:


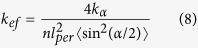


here *n* = *l*_*ef*_/*l*_*per*_ is the amount of “zigzag’s”, and <sin^2^(α/2)> = 0.75 (see supplementary material Table S3).

The angular deformation constant, *k*_*α*_, used in the Treloar *et al.* paper^23^, is also related to the rigidity parameter, *a*. Indeed, according to Treloar *et al.*, the potential energy, *U*, for an angular deformation of amount *δα* is:


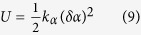


At the same time, the bending energy, *E*_*b*_, of an isotropic macromolecule is


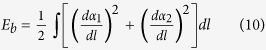


here 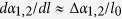 characterize the change in monomer orientations (in two directions, perpendicular to the local chain orientation) along the chain, *l*_0_ is the monomer unit length.

Therefore, the bending energy per one monomer, 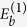, is


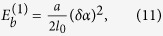


so, we can conclude that


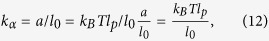



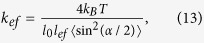


In the case of a system where the coiled structure of gelatin tends to be minimal we have: 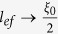 therefore using Equation 8 combined with Equation 6 we have, 
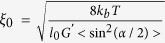
. In the case of a fully cross-linked network (see Fig. 3a) we can hypothesize that the cross-linking mechanism prevents coiled fibrils formation, hence the observed network tends to exhibit almost only thin, semi-flexible chains. Rheology experiments performed on the gel to evaluate its elastic modulus *G*′^24^, can then be used to compare the calculated value for the mesh size of such a network with the one observed with cryo-SEM. Using *G*′ = 9100 Pa, we obtain a value for the network mesh size of *ξ*_*model.hard*_ = 2 × 10^2^ nm, which is in good agreement with the experimental data, *ξ*_*obs.hard*_ = 3.4 ± 0.7 × 10^2^ nm.

In the case of a network mostly composed of coiled fibrils which are not flexible, the effective length of the flexible region would be greatly reduced. The minimum value required to form a bend would be at least two persistence length of the uncoiled flexible chains interconnecting the larger bundles (see Fig. 3b). The persistence length of gelatin is known to be of 2.7 nm^25^, and *l*_*ef*_ = 5.4 nm. Substituting this value into equation (13) above and using the experimental value obtained for the lightly cross linked gel, G′ = 7800 Pa obtained at 25 °C, we obtain, an upper limit for the mesh size of *ξ*_*model.soft*_ ~ 3.7 μm. The mesh size approximated from Fig. 3b (see supplementary section Table S2) is *ξ*_*obs,soft*_ ~ 0.9 ± 0.3 μm. This is consistent with this model considering that the flexible region would be on average slightly longer experimentally.

In conclusion we have shown that the ability to directly image the internal network structure of a hydrogel using cryo-SEM, has enabled us to develop an analytical model which incoporates both the semi-flexible chain strucutre as well as the supra-molecular organization in the thermomechanical response functions of complex biological networks.

## Additional Information

**How to cite this article**: Marmorat, C. *et al.* Cryo-Imaging of Hydrogels Supermolecular Structure. *Sci. Rep.* **6**, 25495; doi: 10.1038/srep25495 (2016).

## Supplementary Material

Supplementary Information

## Figures and Tables

**Figure 1:**
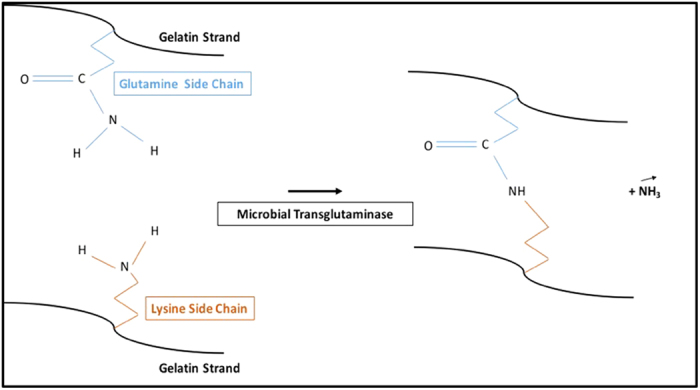
Representation of the iso-peptide bond formation resulting from the catalytic transglutaminase reaction between the glutamine side chain of a gelatin strand and the lysine side chain of another gelatin strand.

**Figure 2:**
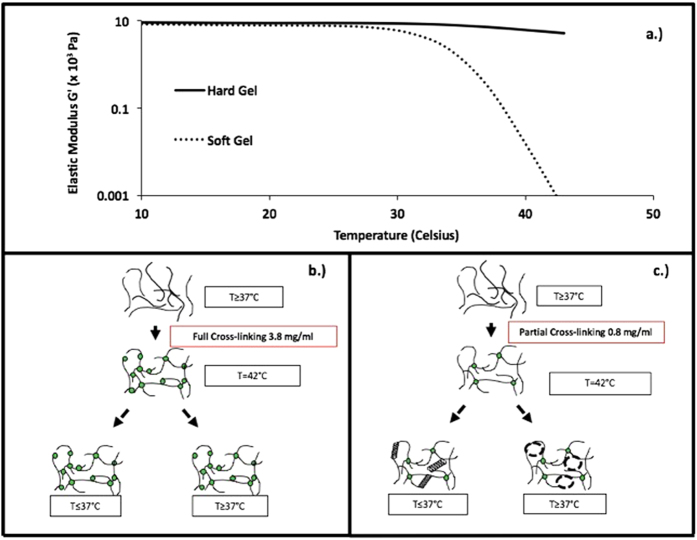
(**a**) Temperature sweep rheograms of the hydrogels at different cross-linking densities. (**b**) Network topology hypothesis for the fully cross-linked gel. (**c**) Network topology hypothesis for the partially cross-linked hydrogel.

**Figure 3:**
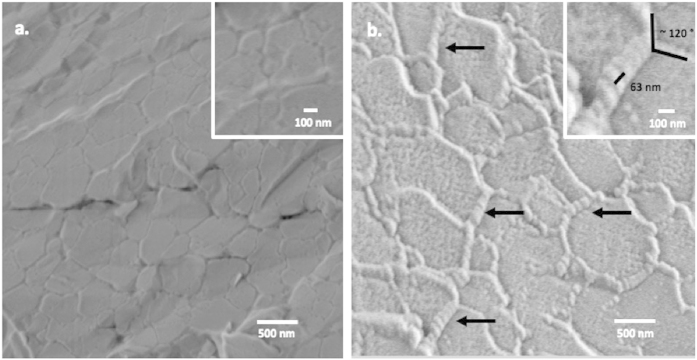
Cryo-SEM pictures of the hydrogels: (**a**) 3.8 mg/ml cross-linking density. (**b**) 0.5 mg/ml cross linking density. The arrows point to the coiled gelatin bundles.

**Figure 4:**
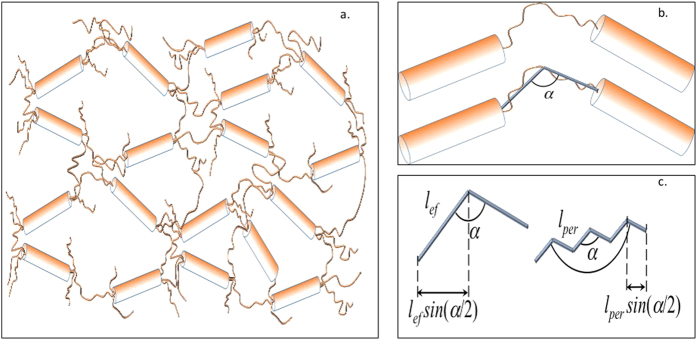
(**a**) Representation of a gelatin based cross linked hydrogel system. (**b**) Connecting angle in the semi-flexible domain of the network. (**c**) Representation of the “bendable” part of the network function of gelatin chains persistence length, *l*_*per*_, and effective length, *l*_*ef*_.
