# Supplementary material for: Cryo-Imaging of Hydrogels Supermolecular Structure
Source: Sci Rep. 2016 May 5;6:25495. doi: 10.1038/srep25495 (PMC4857098; doi:10.1038/srep25495)
Supplement: Supplementary Information [file srep25495-s1.pdf]

Cryo-Imaging of Hydrogels Supermolecular Structure.

Clement Marmorat[1], Arkadii Arinstein[2], Naama Koifman[3],
Yeshayahu Talmon[3], Eyal Zussman[2], Miriam Rafailovich[1]

[1]Department of Materials Science and Engineering, Stony Brook University,New York, USA
[2]Department of Mechanical Engineering, Technion, Haifa, Israel
[3]The Wolfson Department of Chemical Engineering, Technion, Haifa, Israel

SUPPLEMENTARY MATERIAL

Methods:

All water used was deionized using a Milli-Q Integral 10 from Millipore water purifier and filtered through a .22ym Millipak filter from Millipore. All chemicals were purchased from Sigma Aldrich, St Louis, USA unless otherwise notified.

Hydrogels Preparation:

Gelatin, Type A, Bloom 300 was first dissolved at 65°C into water at a concentration of 10% w/v until complete dissolution. The transglutaminase crosslinking agent was purchased from Ajinomoto Foods: Microbial Transglutaminase (MTG Activa TI) and used to make a stock solution into water at a concentration of 10% w/v, each time samples were made. After bringing both the gelatin and the MTG solutions at 42°C they were mixed in the appropriate proportions, poured into P35 petri dishes and incubated for 24 hours at 37°C. After incubation the gels were heated at 65°C for 10 minutes to deactivate the remaining MTG enzyme in the samples. The samples were then rinsed with water and swelled for 24 hours in water. Two gels were made. The first gel was softly cross-linked at a concentration of 0.5mg.ml^{-1} of MTG in gelatin. A harder gel was cross-linked at a concentration of 3.8mg.ml^{-1} of MTG in gelatin. Both gels were casted at a total volume of 3mL.

Rheology:

Rheological measurements were performed on a Discovery DHR-2 rotational rheometer (TA Instruments, USA) under oscillatory shear deformation. The 20 mm parallel-plates Peltier configuration was used with #1200 waterproof sand paper to improve adherence and avoid slipping during deformation of the sample.

Mesh size calculation

The swelling behavior was recorded in order to calculate the molecular weight of polymer between crosslinks as well as the mesh size (or distance between entanglements) of the network. The gels

were first casted following the procedure described above, and their mass, M_r, was recorded as the mass in the relaxed state. Then, after being swelled in excess water for 24 hours at 37°C, their mass, M_{sw} was recorded as the mass of the swelled gel. Then, they were dried at 37°C until reaching a constant mass of dry product M_d. The swelling ratio is defined as

$$Q_m = \frac{M_{sw}}{M_d} \tag{S.1}$$

The polymer volume fraction, $\bar{v}$, in the hydrogel in the swelled state can be calculated:

$$\begin{aligned} \bar{v} &= \frac{M_p/\rho_p}{M_p/\rho_p + M_s/\rho_s} = \frac{M_p/\rho_p}{M_p/\rho_p + (M_{sw} - M_p)/\rho_s} = \\ &= \frac{1/\rho_p}{1/\rho_p + (M_{sw}/M_p - 1)/\rho_s} = \frac{\rho_s}{(Q_m - 1)\rho_p + \rho_s}, \end{aligned} \tag{S.2}$$

here ρ_p and ρ_s are the density of the polymer and of the solvent, respectively; M_p is the mass of the polymer in the swelled samples which assumes to be equal to the mass of dry samples, M_d.

The average molecular weight between cross-links could then be estimated with the help of following equation:

$$\frac{1}{\overline{M}_c} = \frac{2}{\overline{M}_n} - \frac{(V/V_1)\ln(1-\bar{v}) + \bar{v} + \chi_1\bar{v}^2}{\bar{v}^{1/3} - \bar{v}/2}, \tag{S.3}$$

where $\overline{M}_n$ is the average molecular weight of the polymer, V_1 is the molar volume of the solvent, χ_1 is the Flory Huggins interaction parameter, and $\overline{V}$ is the specific volume of the polymer.

Finally we have:

$$\xi = \bar{v}^{-1/3} l_0 \sqrt{2\overline{M}_c C_n / M_r}, \tag{S.4}$$

where $\overline{M}_c$ is the calculated average molecular weight between two neighbor crosslinks, M_r is the molecular weight of the repeating unit (monomer), l_0 is the length of the bond along the polymer backbone average of a C-C bond and two C-N bonds lengths, and C_n is the characteristic ratio of the polymer which can be calculated with the help of the following equation:

$$C_n \sim C_\infty = (2l_{per}/l_s) - 1, \tag{S.5}$$

with $\boldsymbol{l_{per}}$ being the persistence length of the gelatin and $\boldsymbol{l_s}$ is the linear segment length here $\boldsymbol{l_s} = \boldsymbol{l_0}$.

The obtained results are shown in the table below:

Table S.1. Flory-Rhener calculations for mesh size evaluation of the Hard Gel.

	Q_m	$\bar{v}$	$\overline{M}_c$ (Da)	ξ (nm)
**Hard Gel**	8.43	0.080	23149	21.3
**Error**	0.10	9.7×10^{-4}	306	0.22

As far as the mesh size calculation based of the rheological experiments we used the well-known equation $G' = RT/N_A\xi^3$ Rubber Elasticity Theory model to estimate the value of the average mesh size:

$$\xi = \sqrt[3]{\frac{RT}{G'N_A}} \quad \text{(S.6)}$$

where G' being the elastic modulus, N_A is the Avogadro constant, R is the perfect gas constant, and T is the temperature in Kelvin. Using a value for the elastic modulus G' for the fully cross-linked gel shown in Figure 2.a), we obtained that $\xi \approx 6.6$ nm .

Cryo-SEM:

Cryo-SEM imaging was performed at the Israel Institute of Technology, Technion, in Haifa Israel by the Talmon Group on a ZeissUltra Plus HR-SEM equipped with a Bal -Tec cryo-stage allowing imaging of samples close to −150°C. Because hydrogels are mostly made out of water, it is a challenge to image them in their natural conditions. The samples were first mounted on a cryo-SEM sample holder, and then plunged into a freezing liquid ethane bath and then transferred to a liquid nitrogen bath. Once cooled down both sample holder and specimen were transferred to a protected atmosphere shuttle under low temperature and vacuum conditions to prevent contamination in a VT-100 shuttle (Leica). The shuttle was then connected to a Leica EM BAF 060 Freeze-Fracture replication and cryo-SEM sample preparation system in order to expose a fracture profile of the network by impacting the cross section of the specimen with a blade. Finally, the specimen was transferred via the same shuttle to the pre-cooled HR-SEM chamber to be imaged. Samples were imaged without etching or coating at low acceleration voltage in a constant desire to minimally affect the original structure and network properties of the material.

Imaging was first performed on the hardly cross-linked gel and some typical images are presented below:

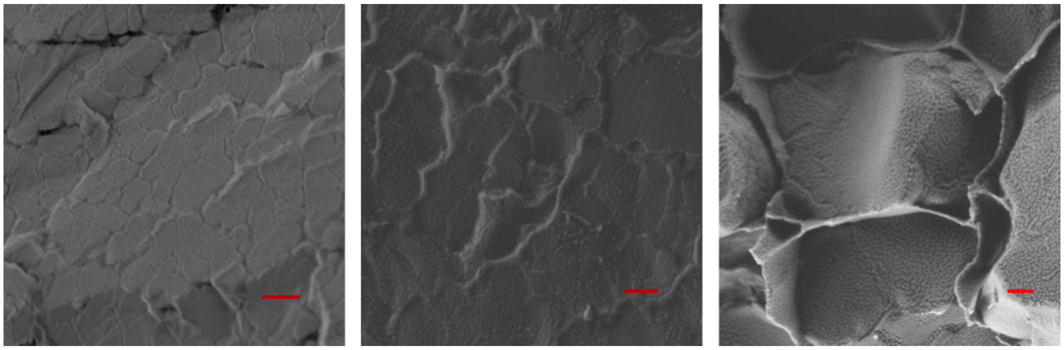

Figure S.1: Evolution of the Hard gel network at 1. *t* = 0min; *t* = 20min and *t* = 1 h 30 min; each scale bar represents 500 nm.

Similarly images were obtained for the softly cross-linked network:

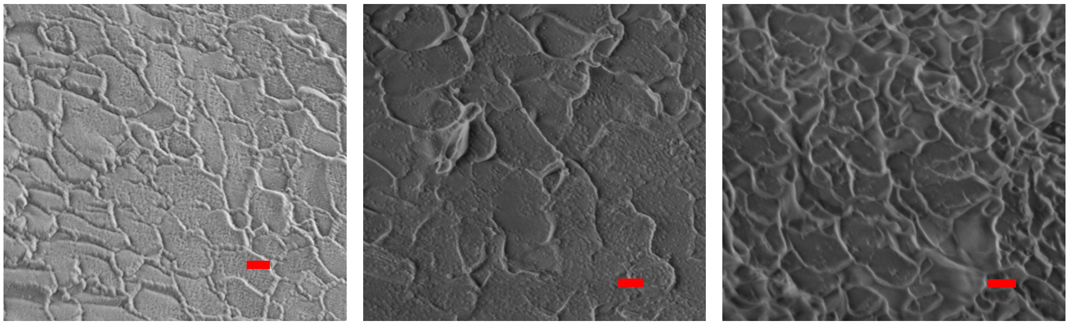

Figure S.2: Cryo SEM images of a 0.5 mg×ml^{-1} cross-linked hydrogel at *t* = 0 min; *t* = 10 min and *t* = 45min. Each scale bar represents 500 nm.

We can notice from the time dependent imaging of the substrates that the network is affected while imaged. Beam etching of the sample during the scan due to poor electric conductivity might be responsible of the rearrangement of the structure.

Mesh size evaluation was performed from the pictures represented in Figure 3. of the main paper. The observed mesh size of the network for both the hardly and softly cross-linked gels are given in the table below:

Table S.2 Supplementary material: Observed mesh size evaluation from cryo-SEM pictures.

Mesh cell	Hard gel : 3.8mg/ml mtg	Soft Gel : 0.5 mg/ml mtg
1.00	0.31	1.00
2.00	0.31	0.64
3.00	0.38	1.09
4.00	0.42	0.73
5.00	0.27	0.91
6.00	0.35	0.91
7.00	0.23	0.82
8.00	0.31	0.82
9.00	0.54	1.00
10.00	0.38	0.73
11.00	0.38	1.64
12.00	0.35	0.55
13.00	0.23	1.00
14.00	0.38	0.73
15.00	0.23	0.55
16.00	0.35	1.36
17.00	0.38	0.91
18.00	0.31	0.36
19.00	0.35	0.91
20.00	0.38	0.82

ξ obs.hard AVERAGE	ξ obs.soft AVERAGE
0.34	0.87
**Standard Deviation**	**Standard Deviation**
0.07	0.28

The angle at which the fibers branch α was evaluated from Figure 3a. and 3b. from the paper. The results of a visual estimation on 10 samples are recorded in the table below:

	Connecting Angle (Degrees)
1	140
2	90
3	120
4	130
5	120
6	170
7	60
8	140
9	70
10	130
**Average**	117
**Standard Deviation**	34

Table S.3 Supplementary material: Statistical analysis of the connecting angle α between fibrils.
